# Sigmar1 regulates endoplasmic reticulum stress-induced C/EBP-homologous protein expression in cardiomyocytes

**DOI:** 10.1042/BSR20170898

**Published:** 2017-07-17

**Authors:** Shafiul Alam, Chowdhury S. Abdullah, Richa Aishwarya, A. Wayne Orr, James Traylor, Sumitra Miriyala, Manikandan Panchatcharam, Christopher B. Pattillo, Md. Shenuarin Bhuiyan

**Affiliations:** 1 Department of Pathology and Translational Pathobiology, Louisiana State University Health Sciences Center, Shreveport, LA 71103, U.S.A.; 2 Department of Molecular and Cellular Physiology, Louisiana State University Health Sciences Center, Shreveport, LA 71103, U.S.A.; 3 Department of Cellular Biology and Anatomy, Louisiana State University Health Sciences Center, Shreveport, LA 71103, U.S.A.

**Keywords:** C/EBP-homologous protein (CHOP), Cardiomyocytes, Endoplasmic reticulum-Stress, Inositol requiring kinase 1a (IRE1a), spliced-X-box binding protein 1 (XBP1s), Sigma 1 receptor (Sigmar1)

## Abstract

C/EBP-homologous protein (CHOP) is a ubiquitously expressed stress-inducible transcription factor robustly induced by maladaptive endoplasmic reticulum (ER) stresses in a wide variety of cells. Here, we examined a novel function of Sigma 1 receptor (Sigmar1) in regulating CHOP expression under ER stress in cardiomyocytes. We also defined Sigmar1-dependent activation of the adaptive ER-stress pathway in regulating CHOP expression. We used adenovirus-mediated Sigmar1 overexpression as well as Sigmar1 knockdown by siRNA in neonatal rat ventricular cardiomyocytes (NRCs); to induce ER stress, cardiomyocytes were treated with tunicamycin. Sigmar1-siRNA knockdown significantly increased the expression of CHOP and significantly induced cellular toxicity by sustained activation of ER stress in cardiomyocytes. Sigmar1 overexpression decreased the expression of CHOP and significantly decreased cellular toxicity in cells. Using biochemical and immunocytochemical experiments, we also defined the specific ER-stress pathway associated with Sigmar1-dependent regulation of CHOP expression and cellular toxicity. We found that Sigmar1 overexpression significantly increased inositol requiring kinase 1α (IRE1α) phosphorylation and increased spliced X-box-binding proteins (XBP1s) expression as well as nuclear localization. In contrast, Sigmar1 knockdown significantly decreased IRE1α phosphorylation and decreased XBP1s expression as well as nuclear transport. Taken together, these results indicate that Sigmar1-dependent activation of IRE1α-XBP1s ER-stress response pathways are associated with inhibition of CHOP expression and suppression of cellular toxicity. Hence, Sigmar1 is an essential component of the adaptive ER-stress response pathways eliciting cellular protection in cardiomyocytes.

## Introduction

Endoplasmic reticulum (ER) is called the protein folding ‘factory’ of the secretory pathway as approximately one-third of the cellular proteome is synthesized, modified, and folded in the ER [1,2]. Virtually all proteins destined for the calcium handling, transmembrane receptors, growth factors, and hormones undergo folding and maturation within the ER, and are then trafficked to various membrane compartments or secreted [1,2]. The ER maintains a unique protein quality control system that allows specialized modifications such as glycosylation and disulphide bond formation that are essential for the correct folding and functioning of these proteins [2-5]. Molecular chaperones and enzymes assist in protein folding by interacting with them, and function as key determinants for their exit from the ER [2]. Terminally misfolded proteins are dislocated across the ER membrane and degraded by the cytosolic ubiquitin–proteasome system, a process called ER-associated degradation (ERAD) [2,6]. The ER protein quality control machinery also regulates a major cell signaling pathway that halts protein biosynthesis, and expands protein folding and degradation capacity in response to proteotoxic stress, a process known as the unfolded protein response (UPR) [2,3,6]. Therefore, efficient folding of newly synthesized proteins as well as protein quality control and degradation are indispensable for protein homeostasis required for normal cellular function [7].

ER function is perturbed by chronic stresses that underlie heart disease; exposure to free radicals, ischemia, hypoxia, elevated protein synthesis, and gene mutation can lead to accumulation of unfolded and misfolded proteins in the ER, a condition referred to as ER-stress [8,9]. ER-stress induces an adaptive response to restore ER homeostasis by reducing the accumulation of unfolded proteins by activating UPR. The traditional ER-stress response involves sensing of calcium and unfolded or damaged proteins in the ER through the calcium binding protein BiP (GRP78), which binds/activates three distinct stress response pathways: protein kinase-like ER kinase (PERK), inositol requiring kinase 1α (IRE1 α), and activating transcription factor 6 (ATF6). These three ER-stress response mediators initiate a cascade of cytoprotective signaling to restore ER homeostasis and promote cell survival that alters protein synthesis and other features of cellular adaptation to stress or protein unfolding and aggregation [6,10,11]. In conditions of prolonged stress, the UPR fails to protect cells against ER-stress and the signaling switches from an adaptive response (prosurvival) to a maladaptive response (cell death) by transcriptional induction of C/EBP-homologous protein (CHOP) [12,13]. Extensive studies suggest that ER-stress-induced apoptosis is the key contributor to the pathogenesis of a series of cardiovascular diseases including ischemia/reperfusion heart diseases [14,15], atherosclerosis [16,17], acute coronary syndrome [18], myocardial infarction [6,19], and heart failure [20,21].

CHOP is a stress-inducible transcription factor ubiquitously expressed at very low levels under basal conditions and its expression is robustly induced by cellular stresses in a wide variety of cells [22-25]. Maladaptive ER-stress leads to induction of CHOP nuclear expression [22-25]. Extensive studies demonstrate that overexpression of CHOP and microinjection of CHOP leads to cell-cycle arrest and/or apoptosis [22-25]. Similarly, excessive activation of CHOP is associated with various cardiovascular diseases, including atherosclerosis, vascular diseases, myocardial reperfusion injury, and heart failure [26-28].

Sigma-1 receptor (Sigmar1) is a ubiquitously expressed molecular chaperone protein in the body [29-37]. Sigmar1 has been reported to reside at the interface between the ER and the mitochondrion (also known as the mitochondrion-associated ER membrane, MAM) [38,39]. Sigmar1 has been found to promote cellular survival of non-cardiomyocytes by regulating certain ER-stress sensors at the MAM under cellular oxidative/ER-stress [38,40-42]. Sigmar1-dependent protective ER-stress response signaling pathways have been reported to be largely dependent on the cell types used for experiments [38,41,43,44]. Though CHOP is known to be one of the highest inducible genes during ER stress [12,23,45-47], Sigmar1’s function in the regulation of CHOP remains unknown.

In the current study, we investigated whether and how Sigmar1 can regulate the ER-stress-induced CHOP expression in cardiomyocytes. We also dissected the Sigmar1-dependent regulation of the specific adaptive ER-stress response pathway to regulate CHOP expression in cardiomyocytes.

## Materials and methods

### Materials

Reagents were obtained from the following sources: DMEM (Gibco), FBS (Gibco), AdEasy system (Agilent Technologies), Lipofectamine 2000 (Invitrogen), OptiMEM (Gibco), Cell Lytic M (Sigma–Aldrich), protease inhibitor cocktail (Roche), precast 7.5–15% Criterion Gels (Bio–Rad), DAPI (Invitrogen), tunicamycin (Sigma–Aldrich), and Vectashield Hardset (Vector Labs, H1400). Tunicamycin (Sigma–Aldrich) was dissolved in DMSO (final concentration: 0.1%). All other chemicals were reagents of molecular biology grade obtained from standard commercial sources.

### Animals

All procedures for handling animals complied with the *Guide for Care and Use of Laboratory Animals* and were approved by the Animal Experimentation Committee of LSU Health Sciences Center-Shreveport. All animals were cared for according to the National Institutes of Health guidelines for the care and use of laboratory animals. Timed pregnant Sprague–Dawley rats were purchased from Charles River Laboratories International, Inc. (Portage, MI).

### Neonatal rat cardiomyocyte isolation and cultures

Primary neonatal rat ventricular cardiomyocytes (NRCs) were isolated from the ventricles of 1–2-day old Sprague–Dawley rat pups as previously described [48,49]; 1.5 × 10^6^ cells per 10-cm plate were grown in DMEM (Gibco) containing 2% FBS (Gibco) and 1% antibiotic-antimyotic (Gibco). Cells were treated with or without tunicamycin 72 h post-infection. All cell culture treatments were repeated in three independent experiments.

### Recombinant adenovirus expressing Sigmar1

We prepared adenoviral constructs containing wild-type Sigmar1 by cloning into a pShuttle-CMV vector; replication-deficient recombinant adenoviruses were made using the AdEasy system (Agilent Technologies) [48,49]. In order to distinguish the expression of exogenous Sigmar1 from endogenous Sigmar1 protein, the Sigmar1 construct was FLAG-tagged at the N-termini. NRCs were infected with adenovirus for 2 h and then replaced with fresh medium. Cells were harvested for 24–72 h post-infection. Infection of parallel plates with adenovirus expressing β-galactosidase served as controls for all the experiments.

### siRNA knockdown of Sigmar1

A pool of siRNAs (Invitrogen) was tested for their capacity to reduce Sigmar1 protein levels in NRCs. We used three sets of siRNAs for Sigmar1 to silence endogenous Sigmar1 expression: siRNA-1 (GGAUCACCCUGUUUCUGACUAUUGU and ACAAUAGUCAGAAACAGGGUGAUCC), siRNA-2 (GGGACGAUACUGGGCUGAGAUUUCA and UGAAAUCUCAGCCCAGUAUCGUCCC), and siRNA-3 (CAGGACUUCCUCACCCUCUUCUAUA and UAUAGAAGAGGGUGAGGAAGUCCUG).

The most potent Sigmar1 silencing siRNA-1 was chosen and used in all the experiments. A non-specific siRNA was used as a negative control in all the silencing experiments. Twenty-four hours after plating, cells were transfected with siRNA and Lipofectamine 2000 (Invitrogen) in OptiMEM (Gibco) medium overnight [48,49].

### Protein extraction and Western blot analyses

To prepare total proteins, NRCs were washed with PBS and the cells were lysed with Cell Lytic M (Sigma–Aldrich) lysis buffer, supplemented with Complete Protease Inhibitor Cocktail (Roche) [48,49]. The lysed cells were homogenized by sonication and centrifuged at 14000×***g*** for 15 min to sediment any insoluble material. The protein content of the soluble lysates was measured using the modified Bradford protocol/reagent relative to a BSA standard curve (Bio–Rad). Protein lysates were separated on SDS/PAGE using precast 7.5–15% Criterion Gels (Bio–Rad) and transferred on to PVDF membranes (Bio–Rad). Membranes were blocked for 1 h in 5% non-fat dried milk and exposed to primary antibodies overnight. The following primary antibodies were used for immunoblotting: anti-Sigmar1 (1:1000, Invitrogen) anti-IRE1α (1:1000, Cell Signaling Technology, Inc.), anti-p-IRE1α (1:1000, Novus Biologicals), anti-CHOP (1:500, Cell Signaling Technology, Inc.), anti-spliced X-box binding protein 1 (XBP1s) (1:200, BioLegend), anti-tubulin (1:1000, Cell Signaling Technology, Inc,), anti-actin (1:10000), anti-Flag (1:1000, Sigma–Aldrich), and monoclonal anti-GAPDH (1:5000, Millipore). Membranes were then washed, incubated with alkaline phosphatase–conjugated secondary antibodies (Santa Cruz Biotechnology), exposed with ECF reagent (Amersham) and finally, detected on a ChemiDoc™ Touch Imaging System (Bio–Rad). Densitometry on scanned membranes was done using ImageJ software (National Institutes of Health, Bethesda, MD, U.S.A.).

### Immunocytochemistry

For immunocytochemistry (ICC) of NRCs, cells were grown on Lab-Tek II chamber slides (Thermo Scientific, 154461) plated at a density of 1 × 10^5^ cells/well. Subsequently, immunofluorescent microscopy was performed essentially as described previously [48-50]. Cells were first washed with PBS, fixed for 15 min in 4% paraformaldehyde, permeabilized with 0.5% Triton-X 100 in PBS, and exposed to an antigen retrieval solution (0.1 M glycine, pH 3.5) for 30 min. The fixed cells were incubated in blocking solution (1% BSA, 0.1% cold water fish gelatin, 0.1% Tween-20, 0.05% sodium azide in PBS) for 1 h. Primary antibody incubation was performed overnight at 4°C, whereas the secondary antibody (1:100) along with counterstains were applied for 1 h at room temperature. The following primary antibodies were used: anti-Sigmar1 (1:100, Invitrogen) anti-actin (1:1000, Sigma–Aldrich), anti-TnI (1:1000, Millipore), anti-CHOP (1:100, Cell Signaling, Technologies, Inc.), α-cardiac actin (1:1000, Sigma–Aldrich), anti-XBP1s (1:200, BioLegend). Coverslips were fixed with Vectashield Hardset (Vector Labs, H1400). Nuclei were stained with DAPI (Invitrogen). Cells were imaged using a Leica TCS SP5 Spectral Confocal Microscope loaded with Leica LAS (AF 2.6.3) and NIS-Elements (AR 4.13.04) software.

### Immunoprecipitation

Co-immunoprecipitation experiments were performed by methods described elsewhere [51]. Briefly, IRE1α antibody (Cell Signaling Technology) conjugated beads were prepared using SureBeads™ protein G magnetic beads according to manufacturer’s instructions (Bio–Rad). Whole cell lysates were prepared from NRCs lysed in lysis buffer consisting of 20 mM HEPES, pH   7.5, 150 mM NaCl, 1% Triton X-100, 10% glycerol, 1 mM EDTA, 10 mM sodium pyrophosphate, 1 mM α-glycerophosphate, 1 mM Na_3_VO_4_, 20 mM NaF, 1 mM phenylmethylsulphonyl fluloride, and 4 mg/ml aprotinin. Whole cell lysates (500 µg of protein) were incubated with the magnetic antibody beads overnight at 4°C on an orbital rotator. Next, the beads were magnetized, supernatants were collected (flow through), and washed with PBS twice. After that, 40 µl of 1× Laemmli buffer was added to beads and incubated for 5 min at 95°C prior to immunoblotting.

### Lactate dehydrogenase release assays

Lactate dehydrogenase (LDH) release was measured by a Cytotoxicity Detection Kit (Roche) as per the manufacturer’s instructions. Color development was measured using a microplate absorbance reader at an absorbance of 492 nm (Bio–Rad) [48].

### Statistics

Data are expressed as mean ± S.E.M. All statistical tests were done with GraphPad Prism software. Data were analyzed using Student’s *t* test (*P*<0.05) for two groups and groups of three or more with one-way ANOVA, followed by Tukey’s *post hoc* test. A value of *P*<0.05 was considered statistically significant.

## Results

### Effects of Sigmar1 knockdown on ER-stress-induced CHOP expression in cardiomyocytes

**Figure 1:**
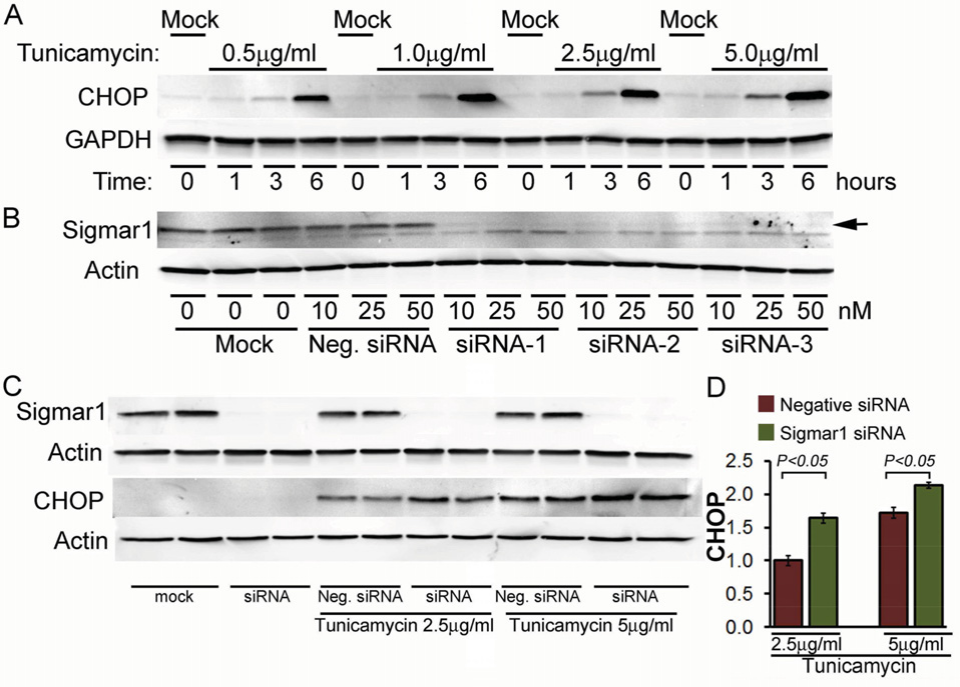
Sigmar1 knockdown increases ER-stress induced CHOP expression in cardiomyocytes (**A**) Western blot showing time- and dose-dependent changes in tunicamycin-induced CHOP expression in cardiomyocytes. (**B**) Western blot showing siRNA knockdown of Sigmar1 in cardiomyocytes. (**C**,**D**) Western blot showing siRNA knockdown of Sigmar1 in cardiomyocytes increased ER-stress induced CHOP expression in NRCs. Bars represent mean ± S.E.M.; *n*=3 experiments.

In the present study, we induced ER-stress in NRCs by treating with tunicamycin at different doses (0.5–5.0 µg/ml) and time points (0–6 h). Tunicamycin acts as a highly specific ER-stress inducer by inhibiting N-linked glycosylation of protein. Western blotting showed time- and dose-dependent increases in CHOP expression in cardiomyocytes (Figure 1A). The expression of CHOP suggested the induction of maladaptive ER-stress induced by tunicamycin treatment in cardiomyocytes. We used siRNA targetted to Sigmar1 to examine the molecular function of endogenous Sigmar1. We tested three different types of Sigmar1 siRNAs. Western blot analysis indicated 90% knockdown of endogenous Sigmar1 in NRCs at 5 days post-transfection with 10 nM of Sigmar1 siRNA. (Figure 1B).

**Figure 2:**
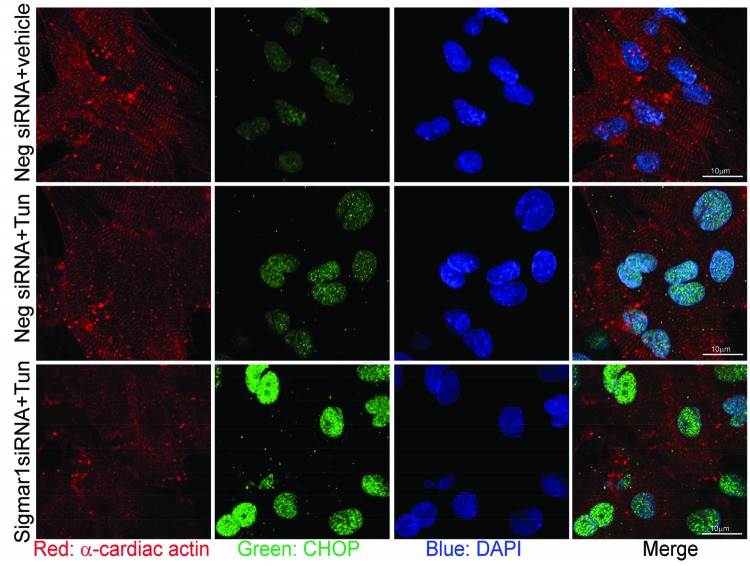
Sigmar1 knockdown increases ER-stress-induced nuclear accumulation of CHOP in cardiomyocytes Confocal microscopic observation of ER-stress-induced CHOP (green) nuclear localization following Sigmar1 knockdown in cardiomyocytes. Cardiomyocytes are counterstained with α-cardiac actin antibody (red) and nuclei with DAPI (blue). *n*=3 experiments.

To define the role of Sigmar1 in ER-stress-induced CHOP expression, we knocked down Sigmar1 in NRCs and then treated the cardiomyocytes with tunicamycin for 6 h. Western blot analysis indicated Sigmar1-siRNA knockdown in NRCs significantly increased CHOP expression in a dose-dependent manner after tunicamycin (both 2.5 and 5.0 µg/ml) treatment (Figure 1C,D). We also observed CHOP nuclear expression by immunofluorescent microscopy. Immunostaining showed minimal expression of CHOP (green) under non-stressed conditions in cardiomycytes (red) (Figure 2). ER-stress induced by treatment with tunicamycin (5.0 µg/ml) for 6 h leads to nuclear accumulation of CHOP. Interestingly, Sigmar1 knockdown further increased the nuclear CHOP accumulation (Figure 2). However, the structure of the sarcomere immunostained by α-cardiac actin was not as clear and intact as the tunicamycin-treated control siRNA group. Future studies are required to demonstrate whether Sigmar1 has any direct role in regulating sarcomere structure or to determine if ER-stress increases the susceptibility to thereof. These data suggest a molecular function of Sigmar1 in cardiomyocytes in regulating CHOP expression in response to ER-stress.

**Figure 3:**
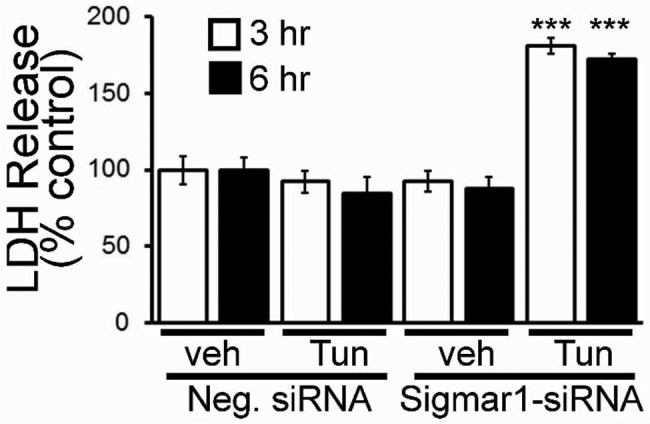
Effect of Sigmar1 knockdown on ER-stress induced cellular damage in cardiomyocytes Tunicamycin-induced LDH release in NRCs. Bars represent mean ± S.E.M.; ***, *P*<0.001 compared with control group by Tukey’s *post hoc* test; *n*=3 experiments.

We also observed cellular damage by measuring LDH release in cells treated with tunicamycin (5.0 µg/ml). Tunicamycin and Sigmar1 knockdown alone have no effect on LDH release. Interestingly, Sigmar1 knockdown significantly increased the susceptibility of ER-stress-induced cellular damage as indicated by significantly increased levels of LDH release (Figure 3). These results indicate that endogenous Sigmar1 is required to protect cardiac myocytes against cellular damage caused by the maladaptive ER-stress response.

### Effects of Sigmar1 overexpression on ER-stress-induced CHOP expression in cardiomyocytes

**Figure 4:**
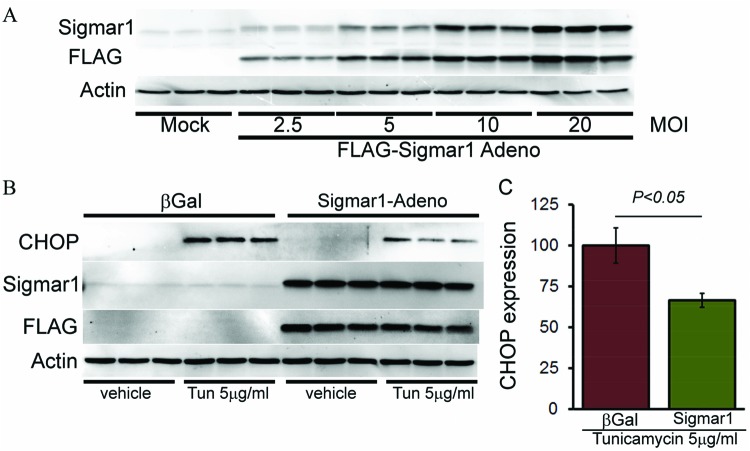
Sigmar1 overexpression decreases ER-stress-induced CHOP expression in cardiomyocytes (**A**) Western blot showing adenovirus-mediated overexpression of Sigmar1 in cardiomyocytes. Representative Western blot (**B**) and densitometric quantitation (**C**) showing Sigmar1 overexpression decreased ER-stress-induced CHOP expression in NRCs. Bars represent mean ± S.E.M.; *n*=3 experiments.

We also performed converse experiments to confirm whether Sigmar1 overexpression can inhibit CHOP expression in response to ER-stress. We generated replication-deficient FLAG-tagged Sigmar1 adenovirus and confirmed expression levels in NRCs by increasing concentrations of adenoviral infections (1–20 multiplicity of infection (MOI)) (Figure 4A). We overexpressed FLAG-Sigmar1 (10 MOI) in NRCs and then treated the Sigmar1 overexpressed NRCs with tunicamycin (5.0 µg/ml, for 6 h) to induce ER-stress. Western blotting revealed increased CHOP expression after tunicamycin treatment and Sigmar1 overexpression significantly decreased CHOP expression in NRCs (Figure 4B,C).

**Figure 5:**
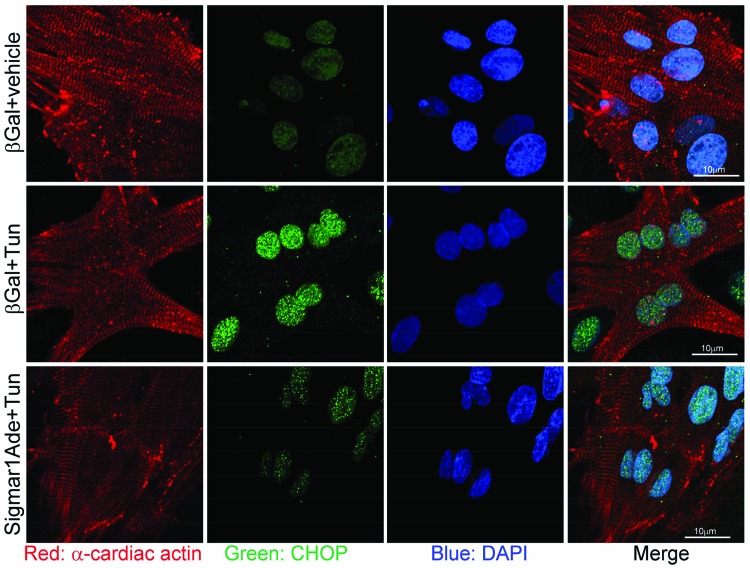
Sigmar1 overexpression decreases ER-stress-induced nuclear accumulation of CHOP in cardiomyocytes Confocal microscopic observation of ER-stress induced CHOP (green) nuclear localization following Sigmar1 overexpression in cardiomyocytes. Cardiomyocytes are counterstained with α-cardiac actin antibody (red) and nuclei with DAPI (blue); *n*=3 experiments.

**Figure 6:**
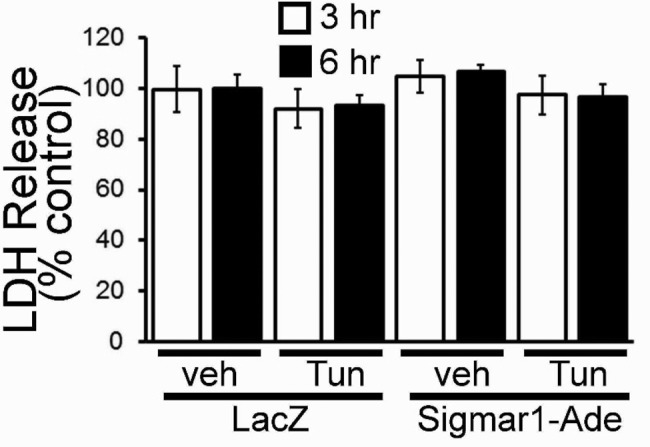
Effect of Sigmar1 overexpression on ER-stress-induced cellular damage in cardiomyocytes Tunicamycin induced LDH release in NRCs. Bars represent mean ± S.E.M.; *n*=3 experiments. Abbreviation: NS, not significant.

We also evaluated CHOP expression by immunofluorescent microscopy. Immunostaining showed nuclear accumulation of CHOP (green) by treatment with tunicamycin (5.0 µg/ml) for 6 h. Notably, Sigmar1 overexpression decreased nuclear CHOP expression (Figure 5). Sigmar1 overexpression did not show any cellular toxicity as measured by LDH release (Figure 6). Together, these data indicate that Sigmar1 overexpression is sufficient to decrease CHOP expression-induced maladaptive ER-stress.

### Sigmar1-dependent activation of the IRE1α pathway of adaptive ER-stress response in cardiomyocytes

**Figure 7:**
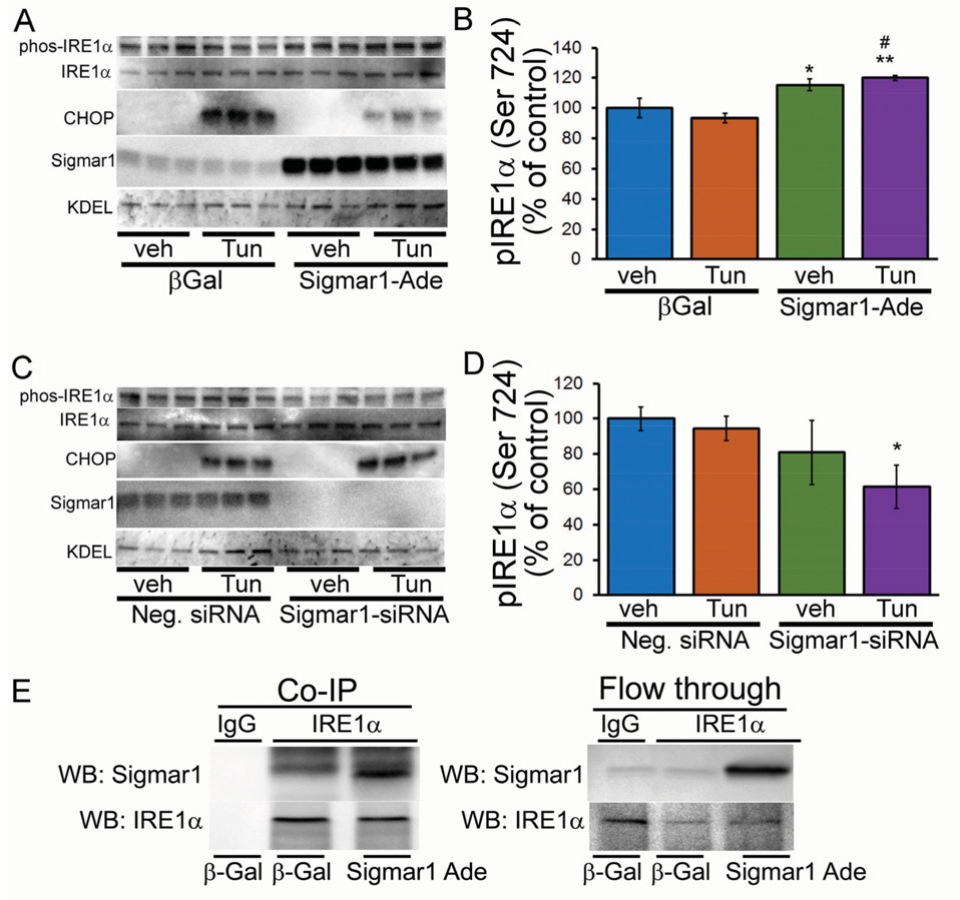
Effect of Sigmar1 expression on ER-stress response pathways (**A**) Effect of adenoviral-mediated Sigmar1 overexpression on tunicamycin-induced changes in expression of proteins involved in the ER-stress response pathway and (**B**) densitometric quantitation of p-IRE1α. (**C**) Effect of Sigmar1 knockdown by siRNA transfection on tunicamycin-induced changes in the expression of adaptive ER-stress proteins and (**D**) densitometric quantitation of p-IRE1α. Bars represent mean ± S.E.M.; *n*=3 experiments. *, *P*<0.05 and **, *P*<0.01 compared with control group; ^#^, *P*<0.05 compared with control tunicamycin treatment group by Tukey’s *post hoc* test. (**E**) Co-immunoprecipitation (Co-IP) studies showing IRE1α interaction with Sigmar1 in cardiomyocytes. Whole cell extracts from Sigmar1 adenoviral overexpressed (Sigmar1 Ade) or control infected (β-Gal) cardiomyocytes were immunoprecipitated with anti-IRE1α antibody. The abundance of immunoprecipitated Sigmar1 was detected by Western blotting using anti-Sigmar1 antibody.

Next, we performed experiments to define the ER-stress pathway that is regulated by Sigmar1 in cardiomyocytes following ER-stress. Amongst the three ER-stress response pathways, IRE1α signaling is known to inhibit CHOP activation and sustained activation of IRE1α signaling enhances cell proliferation and cell survival [35-37]. Moreover, a recent study suggested that Sigmar1 transiently interacts with IRE1α in response to ER-stress, which activates IRE1α by subsequent dimerization/phosphorylation [41]. To further define the involvement of Sigmar1 in the regulation of specific ER-stress response pathways, we overexpressed Sigmar1 in cardiomyocytes by adenoviral overexpression and then treated the cell with tunicamycin to induce ER-stress. Western blot analysis showed that Sigmar1 overexpression significantly increased the phosphorylation of IRE1α (Figure 7A,B). The increase in IRE1α activation is associated with a decrease in CHOP expression by treatment with tunicamycin. We also performed the converse experiment to confirm that Sigmar1 knockdown can inhibit IRE1α phosphorylation. Sigmar1-siRNA knockdown in cardiomyocytes significantly decreased the phosphorylation of IRE1α under conditions of ER-stress (Figure 7C,D). To further confirm whether Sigmar1 may preferentially bind IRE1α and regulate IRE1α phosphorylation, we performed co-immunoprecipitation studies using an anti-IRE1α antibody. Co-immunoprecipitation studies showed that Sigmar1 interacts with IRE1α (Figure 7E). These data suggest that Sigmar1 directly interacts with IRE1α and activates the IRE1α pathway following tunicamycin-induced ER-stress in cardiomyocytes.

### Sigmar1-dependent regulation of XBP1s nuclear localization in cardiomyocytes

**Figure 8:**
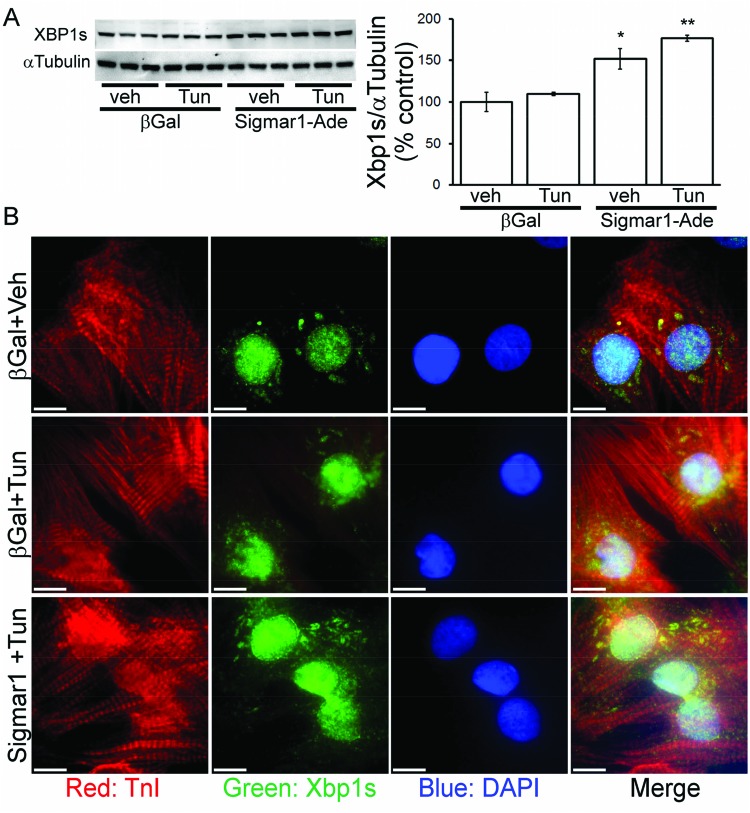
Effect of Sigmar1 overexpression on XBP1s expression in NRCs (**A**) Sigmar1 overexpression increases the expression of XBP1s in tunicamycin treated NRCs. Bars represent mean ± S.E.M.; *n*=3 experiments. *, *P*<0.05 and **, *P*<0.01 compared with control group by Tukey’s *post hoc* test. (**B**) Confocal microscopic observation of the changes in XBP1s (green) nuclear localization by Sigmar1 overexpression in tunicamycin treated cardiomyocytes (cardiac troponin I: red; DAPI: blue).

We established that Sigmar1 regulates the IRE1α pathway of the adaptive ER-stress responses in cardiomyocytes. Upon activation through ER-stress, IRE1α mediates selective splicing of the transcription factor XBP1s leading to the expression of a more stable and active form of XBP1s. Our next set of experiments examined if Sigmar1 can promote cellular protective effects through the IRE1α-XBP1s signaling pathway. We found that Sigmar1 overexpression increased the expression of XBP1s following tunicamycin treatment in cardiomyocytes (Figure 8A). In addition to the Western blot data, we also used immunostaining to demonstrate increased XBP1s nuclear localization in response to Sigmar1 overexpression in cardiomyocytes under conditions of ER-stress. Sigmar1 overexpression caused enhanced XBP1s nuclear localization, which confirms the activation of the IRE1α-XBP1s signaling pathway (Figure 8B). Therefore, Sigmar1 overexpression induced XBP1s expression and nuclear localization is associated with decreased expression and nuclear localization of CHOP in cardiomyoctyes.

**Figure 9:**
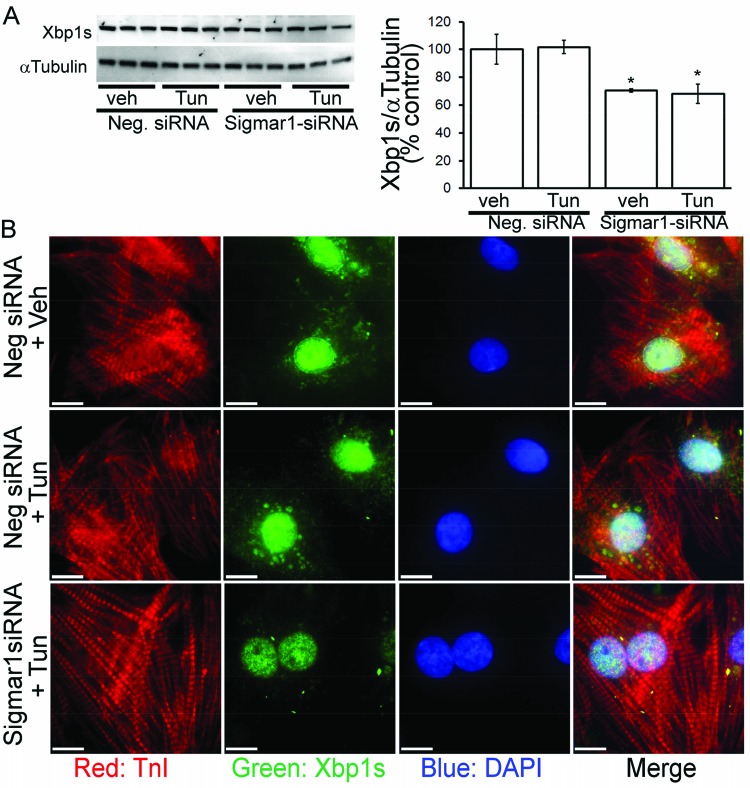
Effect of Sigmar1 siRNA knockdown on XBP1s expression in NRCs (**A**) Sigmar1 knockdown by siRNA transfection decreases the expression of XBP1s in tunicamycin-treated NRCs. Bars represent mean ± S.E.M.; *n*=3 experiments. *, *P*<0.05 compared with control group by Tukey’s *post hoc* test. (**B**) Confocal microscopic observation of the changes in XBP1s (green) nuclear localization by Sigmar1-siRNA knockdown in tunicamycin-treated cardiomyocytes (cardiac troponin I: red; DAPI: blue).

We then examined the effect of Sigmar1-RNA knockdown and found significantly decreased expression of XBP1s following ER-stress in cardiomyocytes (Figure 9A). ICC also confirmed the decreased nuclear localization of XBP1s in the Sigmar1-knockout cardiomyocytes following ER-stress (Figure 9B). Taken together, these results indicate that the Sigmar1-dependent activation of IRE1α promotes the XBP1s nuclear localization that attenuates ER-stress-induced CHOP expression in cardiomyocytes.

## Discussion

We have been exploring the molecular function of Sigmar1 in the heart. Sigmar1’s function in the cardiovascular system is evident by studies showing Sigma receptor ligands alter contractility, calcium influx, and rhythmic activity in cardiomyocytes [52-55]. Despite widespread information on modulation of heart function by Sigmar1 ligands, no previous study has defined the molecular function of Sigmar1 under ER-stress conditions in cardiomyocytes. We previously documented Sigmar1’s expression and signaling in the heart [29-33], kidney [34], and aorta [31,32]. In the present study, we defined a novel function of Sigmar1 in regulating CHOP expression, and defined Sigmar1-dependent-specific ER-stress response pathways involved in CHOP regulation under conditions of ER-stress in the cardiomyocytes. This is the first study, to our knowledge, to demonstrate Sigmar1-dependent regulation of CHOP expression in cardiomyocytes. Additionally, for the first time, we directly showed Sigmar1-dependent activation of the IRE1α-XBP1s signaling pathway in association with decreased CHOP expression in cardiomyoctyes.

CHOP functions as an inducible transcription factor with extremely low expression in healthy cardiomyocytes. Enhanced CHOP expression in the nucleus are canonically up-regulated during cellular toxicity induced by ER-stress. Experimentally, ER-stress can be induced by perturbing the ER environment by chemical substances, such as DTT, thapsigargin, or tunicamycin, which alter redox status, calcium levels, and protein glycosylation in the ER, respectively [56-58]. Treating the cells with any of these compounds results in impairment in ER protein folding and the accumulation of misfolded, and dysfunctional proteins signal the initiation of ER-stress [58,59]. Amongst these, tunicamycin is a specific inhibitor of N-linked glycosylation, which occurs in the ER, and is a highly specific inducer of ER-stress. In this manuscript, we implemented a genetic approach using Sigmar1 overexpression and siRNA knockdown in cardiomyocytes to define the direct effect of Sigmar1 in modulating ER-stress responses as well as CHOP expression under ER-stress condition in cardiomyocytes. Sigmar1 overexpression significantly increased IRE1α expression under ER-stress conditions in cardiomyocytes. We also found that Sigmar1 overexpression was sufficient to increase the expression as well as nuclear transportation of XBP1s and thereby significantly decreased CHOP expression. In contrast, Sigmar1 knockdown decreased IRE1α expression, decreased XBP1s expression as well as nuclear transport, increased the expression of nuclear CHOP and significantly increased cellular toxicity. Our findings are supported by a recent study showing XBP1s can inhibit ER-stress-mediated apoptosis in chondrocytes and chondrosarcoma cells by regulating CHOP expression. Tunicamycin-induced CHOP expression and nuclear translocation was increased by XBP1s siRNA knockdown and were decreased after XBP1s overexpression [60]. In addition, liver-specific XBP1-knockout mice also showed increased CHOP expression and enhanced apoptotic injury in response to tunicamycin-induced ER-stress [61]. These results demonstrate that Sigmar1 can function as a protective ER-stress regulator through a mechanism involving activation of IRE1α-XBP1s in cardiomyocytes.

Excessive activation of UPR and/or ER-initiated apoptosis have been implicated in the pathophysiology of various cardiovascular diseases such as cardiac hypertrophy, ischemia/reperfusion injury of the heart, ischemic heart diseases, atherosclerosis, alcoholic cardiomyopathy, autoimmune cardiomyopathy, myocardial infarction, and heart failure [62]. The UPR elicits paradoxical outputs, inducing cytoprotective functions by establishing homeostasis and cell destructive functions by promoting apoptosis. Amongst the ER-stress-response pathways, sustained activation of IRE1α signaling enhanced cell proliferation and cell survival [35-37]. IRE1α is a transmembrane protein localized in the ER having both kinase and RNase domains. Activation of IRE1α involves its dimerization, oligomerization, and *trans*-autophosphorylation leading to a conformational change that activates the RNase domain [9,63]. Upon stimulation by ER-stress, IRE1α mediates selective splicing of the transcription factor XBP1s leading to the expression of a more stable and active form of XBP1s (spliced form) [63,64]. Then, XBP1s transactivate a subset of target genes involved in protein folding, ER-associated protein degradation, protein translocation to the ER and protein secretion to recover ER function [63]. XBP1s have been reported to inhibit the expression of ER-stress-mediated apoptosis signaling pathway by inhibiting CHOP nuclear expression under conditions of ER-stress [60]. We have demonstrated that Sigmar1 overexpression is sufficient to induce IRE1α phosphorylation, increased the expression of XBP1s and enhanced nuclear localization of XBP1s. Therefore, our data showed that Sigmar1 directly activates the IRE1α-XBP1s pathway of the adaptive ER-stress response pathway and inhibits the expression of CHOP in cardiomyocytes.

In summary, we demonstrated that Sigmar1 is an essential component of the adaptive ER-stress-response pathway eliciting cellular protection in cardiomyocytes from maladaptive ER-stress. Using Sigmar1 overexpression and knockdown in cardiomyocytes, we found Sigmar1-dependent regulation of CHOP expression in association with activation of the IRE1α-XBP1s pathway of the ER-stress response. The IRE1/Xbp1s branch is the most highly conserved arm of the UPR in mammals that functions to resolve proteotoxic stress and restores ER homeostasis in multiple cell types under various conditions [15,65]. A recent study using a combination of gain- and loss-of-function strategies demonstrated that Xbp1s induction protects the heart from myocardial infarction *in vivo* and serves to preserve myocyte viability and contractile function [65]. Moreover, Xbp1s is also reported to be a powerful transcription factor, targetting an array of ER chaperones and a group of molecules involved in ERAD [2,15,65]. Therefore, Sigmar1-dependent activation of IRE1α-XBP1s pathway have a huge therapeutic potential under pathological conditions in the heart associated with protein misfolding. Future studies addressing the direct roles of Sigmar1 in specific ER-stress-response pathways as well as pathway-specific gene activation programs *in vivo* in various pathological conditions are required to better understand the specific functions of each, as determinants of cell survival and tissue damage.

## Abbreviations

BIP: Binding immunoglobulin protein
CHOP: C/EBP-homologous protein
DMEM: Dulbecco's modified eagle medium
C/EBP: CCAAT-enhancer binding proteins
CMV: Cytomegalovirus
ER: endoplasmic reticulum
ERAD: endoplasmic reticulum-associated degradation
GRP78: 78 kDa glucose-regulated protein
ICC: immunocytochemistry
IRE1α: inositol requiring kinase 1α
LDH: lactate dehydrogenase
MAM: mitochondrion-associated ER membrane
MOI: multiplicity of infection
NRC: neonatal rat ventricular cardiomyocyte
Sigmar1: sigma 1 receptor
UPR: unfolded protein response
XBP1: spliced X-box binding protein 1
